# Nano silver and nano zinc-oxide in surface waters – Exposure estimation for Europe at high spatial and temporal resolution

**DOI:** 10.1016/j.envpol.2014.10.022

**Published:** 2015-01

**Authors:** Egon Dumont, Andrew C. Johnson, Virginie D.J. Keller, Richard J. Williams

**Affiliations:** Centre for Ecology & Hydrology (CEH), Maclean Building, Benson Lane, Wallingford, OX10 8BB, United Kingdom

**Keywords:** Engineered nano-particles, Surface waters, Exposure modeling, Hydrological modeling, EU

## Abstract

Nano silver and nano zinc-oxide monthly concentrations in surface waters across Europe were modeled at ∼6 × 9 km spatial resolution. Nano-particle loadings from households to rivers were simulated considering household connectivity to sewerage, sewage treatment efficiency, the spatial distribution of sewage treatment plants, and their associated populations. These loadings were used to model temporally varying nano-particle concentrations in rivers, lakes and wetlands by considering dilution, downstream transport, water evaporation, water abstraction, and nano-particle sedimentation. Temporal variability in concentrations caused by weather variation was simulated using monthly weather data for a representative 31-year period. Modeled concentrations represent current levels of nano-particle production. Two scenarios were modeled. In the most likely scenario, half the river stretches had long-term average concentrations exceeding 0.002 ng L^−1^ nano silver and 1.5 ng L^−1^ nano zinc oxide. In 10% of the river stretches, these concentrations exceeded 0.18 ng L^−1^ and 150 ng L^−1^, respectively. Predicted concentrations were usually highest in July.

## Highlights

• We modeled nano-particle concentrations in surface waters across Europe.
• Modeled concentrations are generally highest where population densities are high.
• Expected levels exceed 0.18 ng L^−1^ nano silver in 10% of the river reaches.
• Expected levels exceed 150 ng L^−1^ nano zinc oxide in 10% of the river reaches.
• Modeled concentrations are usually highest during the months June, July or August.

## Teaser

Ten percent of European river stretches have expected concentrations exceeding 0.18 ng L^−1^ nano silver and 150 ng L^−1^ nano zinc-oxide.

## 1 Introduction

Nanotechnology is a major growth industry, and in a review of emerging risks to UK biodiversity, nanotechnology was classified as the highest potential threat (Sutherland et al., 2008). Currently, one of the main sectors in the nanotechnology market is connected with the use and application of engineered nano-particles (ENPs). Concern has arisen about the potential risks posed by the use of these ENPs in consumer products largely because of the uncertainties that exist about the fate and toxicity of ENPs in the environment (Royal Commission on Environmental Pollution, 2008). There are also high uncertainties in the release of ENPs to the environment (e.g. Sun et al., 2014). Therefore improving the understanding of the release and fate of nanotechnology-based products over their life cycle is needed. Because ENPs are used in several types of products they will need to be regulated by environmental frameworks. Within Europe, the regulation on Registration, Evaluation, Authorization and restriction of CHemicals (REACH) is the principal legislative means through which the potential effects of chemicals in products are regulated and controlled. The fact that the potential risk of ENP based products should be addressed under REACH and also, if applicable, other directives for pharmaceutical, biocides, veterinary medicines and plant protection products, means that it is essential that such legislative procedures are fit for purpose. The current standard model prescribed by REACH for assessing environmental concentrations is EUSES. This model only considers removal processes applicable to dissolved chemicals. However, ENPs are not dissolved but suspended. Therefore, some important ENP removal processes are not included in EUSES. These include settling loss, and irreversible transformations to non-nano forms such as ions and large agglomerates (Quik et al., 2011). However, there is a general consensus that exposure assessment of ENPs under REACH would require knowledge on fluxes or rates, instead of distribution coefficients on which conventional models for chemicals are often dependent (Foss Hansen et al., 2011). For these reasons, it is necessary to develop a more appropriate approach to model the fate of ENPs in the environment.

This paper tries to quantify the fate of ENPs in surface waters. The described work was done within the European Union project NanoFATE, which aimed at quantifying the environmental risk posed by the household use of ENP containing products. A number of multi-media and multi-compartment modeling studies assessing ENPs in surface waters have been published in the scientific literature (Boxall et al., 2007, Gottschalk et al., 2009, Mueller and Nowack, 2008, Sun et al., 2014). These studies reported worst-case and sometimes expected estimates of ENP concentrations on national to continental scales. However, these studies do not reflect the reality that exposure to ENPs may be highly variable in both space and time, due to for example spatial variability in population density and temporal variability in river water discharge. More recently, Gottschalk et al. (2011) used for the first time a spatially and temporally explicit method to model ENP (Ag, ZnO, and TiO_2_) concentrations. Their results, which cover Switzerland, show that variations in time, or location, can result in concentration differences in rivers of up to a factor of 1000.

This paper describes the use of the GWAVA model (Dumont et al., 2012, Meigh et al., 1999) to simulate expected concentrations of two ENPs likely to be emitted to surface waters on a widespread basis: nano silver (Ag) and nano zinc oxide (ZnO). Nano Ag is often used for its biocidal properties and is included in a range of personal care products (cosmetics, wound dressings), textiles, paints and other surface coatings, whereas nano ZnO is used in paints and cosmetics, especially sunscreens (Lanzano et al., 2006, VROM, 2008). Modeled concentrations of these ENPs under a worst-case scenario are also described. The GWAVA model has been previously applied across Europe for a range of other aquatic pollutants (Dumont et al., 2012, Johnson et al., 2013).

This study attempts to improve the ability to assess exposure to ENPs. In particular, the approach is temporally explicit for all water bodies, instead of only those with measured river flow time series. In addition to rivers, the model also covers lakes, reservoirs and wetlands. The modeled loss of ENPs from surface waters is based on particle-specific properties. Finally, the scope of the modeling covers the whole of Europe, making it more relevant for ENP risk assessment within REACH.

## 2 Methods

**Fig. 1:**
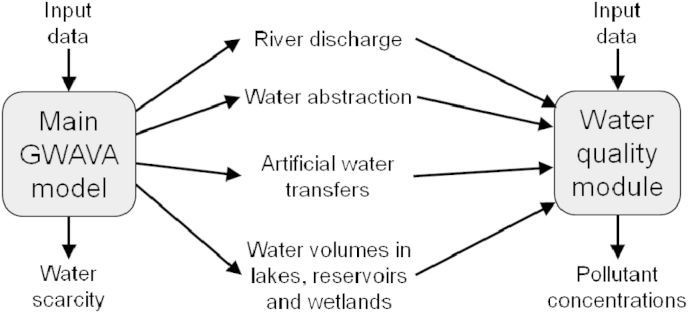
GWAVA overview. Input data are detailed in Table 1.

GWAVA's main component simulates river discharge and a number of other hydrological variables, such as lake water volumes and human water abstractions, in a spatially and temporally explicit manner. In order to model ENP concentrations, GWAVA has a water quality module (Fig. 1) in which the most important input is ENP loading to surface waters. Other inputs to GWAVA are listed in Table 1. For this study, GWAVA was used to model concentrations at a 5′ by 5′ (∼6 by 9 km) spatial resolution,^1^ and a monthly temporal resolution. It is important to clarify that this study is predicting surface water concentrations of ENPs that, whilst still being below 100 nm, may have been transformed after the production stage. Thus transformations that do not make the particle size increase beyond 100 nm nor to individual molecules are not considered as ENP losses. Often such transformations will include sulfidization, phosphatation (Ma et al., 2013a, Ma et al., 2013b), and reversible sorption to suspended material, as opposed to e.g. aggregation and dissolution which usually cause substantial size changes.

**Table 1:** Spatially explicit inputs to the GWAVA model.

Input data	Resolution	Source
Sub-grid elevation distribution^a^	30″	HydroSHEDS (Lehner et al., 2008), GTOPO (USGS, 1996)
Locations of irrigated crop types and the start and end of their growing season	5′	MIRCA2000 (Portmann et al., 2010)
Crop characteristics and growth stage durations for 47 irrigated crop types	Monthly, 5′	Allen et al. (1998), Siebert and Döll (2010), MIRCA2000 (Portmann et al., 2010)
Hydrography	n.a. (vector data)	CCM2.1 (Voght et al., 2007)
Soil texture	5′	HWSD (FAO et al., 2009)
Land cover	5′	GLCC (USGS, 2001)
Climate parameters	10′, monthly	CRU TS 1.2 (Mitchell et al., 2004)
Climate parameters	30′, monthly	CRU TS 2.1 (Mitchell and Jones, 2005)
Lake, reservoir and wetland parameters	5′	GLWD (Lehner and Döll, 2004)
Fraction of water extracted from groundwater	Country	Aquastat (FAO), Eurostat (European Commission, 2010)
Urban, rural, and industrial water demand per capita	Country	Eurostat (European Commission, 2010)
Rural population^b^	5′	FAO (Salvatore et al., 2005)
Total population^b^	2.5′	GPW (Balk and Yetman, 2004)
Cattle, sheep and goat population	0.05°	Wint and Robinson (2007)
% households connected to sewerage^b^	Country	Williams et al. (2012)
Sewage effluent locations and sizes^b^	n.a. (point data)	EEA (2012)

a Used for calculating river depth during the simulation of ENP transport.

b Used for the modeling of ENP loading from point sources.

### 2.1 Nano-particle loading to surface waters

The GWAVA input on ENP loading to surface waters is a gridded map (5′ × 5′ resolution) of loadings (kg km^−2^ year^−1^) prepared individually for each ENP. These grids were prepared in two steps: (1) calculation of ENP loading entering sewage treatment plants (STPs), and (2) calculation of ENP loadings in STP effluents discharged to surface waters. The first step is summarized by the following equation which is applied to each grid cell in Europe:

(1) $$L_{\text{cell}}=L_{P}\cdot P_{\text{cell}}$$

Here, *L*_cell_ is ENP loading to sewage in a specific grid cell (kg year^−1^), *L*_*P*_ is the ENP loading per person (kg person^−1^ year^−1^), and *P*_cell_ is the number of persons whose household is connected to STPs discharging to rivers in the current grid cell.

The value of *L*_*P*_ is estimated by dividing the EU-total ENP loading to STPs (kg year^−1^) by the population of EU27. The EU-total ENP loading to STPs used here was 1.05 million kg year^−1^ for nano ZnO and 8.85 thousand kg year^−1^ for nano Ag. These are the most probable loading values according to Sun et al. (2014). The population of EU27 in 2013 is 503 million persons according to Eurostat (EEA, 2012). Thus the ENP loading per person entering STPs in EU27 is 2.1 g year^−1^ person^−1^ for nano ZnO and 0.018 g year^−1^ person^−1^ for nano Ag. ENP production volumes for nano Ag, on which such ENP release to STPs in the EU can be based, have been estimated by several studies (reviewed by Sun et al., 2014). They vary from to 15 μg person^−1^ year^−1^ for the World (Mueller and Nowack, 2008) to 405 mg person^−1^ year^−1^ for Switzerland (Mueller and Nowack, 2008). Nano ZnO production volumes in the peer-reviewed literature (Gottschalk et al., 2009, Piccinno et al., 2012, Sun et al., 2014) range from 0.11 to 3.2 g person^−1^ year^−1^. The production estimate on which the nano Ag loading to STPs used here was based (64 mg person^−1^ year^−1^) is in the middle of the range previously mentioned, whereas the production estimate for nano ZnO (3.2 g person^−1^ year^−1^) was in the higher end of the literature range. We chose to use values from Sun et al. (2014) because they provide an estimate of loading to STPs arising from the use of ENPs in everyday consumer products. Moreover, this estimate uses the most recent ENP production and behavior information. Also, as opposed to most other estimates, it incorporates a comprehensive spectrum of household applications of nano Ag and nano ZnO, and it uses a large number of data sources regarding ENP production.

Values for *P*_cell_ (Eq. (1)) were based on an extensive dataset (2009–2010 information) describing individual sewage discharge points across Europe (EEA, 2012). These values were modeled in countries not covered by EEA (2012): Poland, Sweden, Switzerland, Croatia, Serbia, Macedonia, Albania, Montenegro, and Bosnia and Herzegovina. This was done by assigning each of the people in a population map for 2013 (based on Balk and Yetman, 2004) to a modeled water course. Subsequently the fraction of people not connected to sewerage (data from Williams et al., 2012) was removed.

The result of Eq. (1) was used to calculate the area-specific loading of ENPs to surface waters through sewage effluent ($L_{\text{cell}}^{*}$ in kg km^−2^ year^−1^):

(2) $$L_{\text{cell}}^{*}=\frac{L_{\text{cell}}}{A_{\text{cell}}}\cdot \left(1-STP_{\text{rem}}\right)$$

Here, *A*_cell_ is the cell area (km^2^), *STP*_rem_ is the EU27-average fraction of ENPs that are removed in STPs. STPs divert most of the ENPs to sludge which is removed and does not reach the effluent. We based the value of *STP*_rem_ on literature reporting either measurements on real STPs, realistic laboratory simulations of STPs, or realistic computer model simulations of STPs (Table S1). For nano Ag, *STP*_rem_ is 0.93 which is the average of ten literature values (Kägi et al., 2011, Kiser et al., 2010, Li et al., 2013, Lombi et al., 2013, Park et al., 2013, Schlich et al., 2013, Sun et al., 2014, Tiede et al., 2010, Wang et al., 2012) ranging from 0.85 to 0.99. For nano ZnO, *STP*_rem_ is 0.84 which is the average of three literature values (Environment Agency, 2011, Lombi et al., 2012, Sun et al., 2014) ranging from 0.81 to 0.88. The lowest values found in the literature were used for *STP*_rem_ in the worst-case scenario (Table 2): 0.85 for nano Ag from Lombi et al. (2013), 0.81 for nano ZnO from Environment Agency (2011).

**Table 2:** Values of parameters *STP*_rem_ (fraction of ENPs that are removed in STPs) and *k* (first-order loss coefficient for ENPs in surface waters) in the two modeled scenarios.

Scenario	ENP	*STP*_rem_ (fraction)	*k* (s^−1^)
Expected	Nano ZnO	0.84	1.26∙10^−5^
Worst-case	Nano ZnO	0.81	0
Expected	Nano Ag	0.93	1.26∙10^−4^
Worst-case	Nano Ag	0.85	0

It is assumed that households are the only source of ENP loading from sewage effluent ($L_{\text{cell}}^{*}$). This is in line with a survey done by Piccinno et al. (2012) which indicated that nano Ag and nano ZnO almost only have household applications.

ENP loading from sewage effluent ($L_{\text{cell}}^{*}$) is assumed constant in time and represents the current situation. Temporal variability of ENP exposure in surface waters was simulated using modeled temporal variability in hydrology over a representative 31-year climate period, as explained in the next section.

### 2.2 Modeling nano-particle transport and loss in surface waters

The loading of ENPs to surface water (lakes, rivers, wetlands, and/or reservoirs) is simulated using $L_{\text{cell}}^{*}$ described in the previous section. Simultaneously, the ENPs are routed down the river network, during which concentrations are calculated by accounting for any ENP losses and dilution by river discharge. Spatio-temporal variation in river discharge was modeled using monthly observed weather data for the period 1970–2000, and has a good fit with discharge measurements across Europe (Dumont et al., 2012, Gudmundsson et al., 2012). The 1970–2000 period was used because it had many available observations of weather and river discharge, and the spatio-temporal variability in this period is very similar to the current situation. The duration of this period (31 years) is long enough to fully characterize the variability of all used climate parameters, and so the modeled water discharge should provide a good estimate of the likely range that would be seen in the rivers across Europe. Loss of ENPs with abstracted water (e.g. for irrigation) was accounted for. Increase in concentrations due to water evaporation was also modeled. Based on research with nano CeO_2_ (Quik et al., 2012) and a literature review for a range of different ENPs (Quik et al., 2011), ENP loss in river water follows first-order kinetics. ENP loss was therefore modeled as a first-order process:

(3) ⅆC/ⅆt=−k·C+f(C,t,cell)

Where *C* is any ENP concentration (kg m^−3^) in any modeled surface water at any modeled time, *t* is time (s), *k* is a first-order loss coefficient (s^−1^) characterizing ENP sedimentation and dissolution in surface water, and *f* (*C*,*t*,cell) is the impact of modeled variables that are not specific for ENPs (kg m^−3^ s^−1^) such as hydrological variables which vary with *C*, *t* and grid cell (cell). This impact of variables that are not ENP-specific is described in detail in Dumont et al. (2012). Equation (3) implies negligible contribution of homo aggregation (ENPs aggregating with each other) to ENP loss, since homo aggregation is known to behave as a second-order process (Areepitak and Ren, 2011, von Smoluchowski, 1912). Thus Equation (3) is consistent with the usually very low aquatic concentrations which make it unlikely that ENPs of the same type collide frequently with each other (Praetorius et al., 2012). Also Equation (3) implies that more ENP is transformed when the residence time in water is longer. Hence the importance of modeling lakes and wetlands. The resulting model output consists of aquatic ENP concentrations in each modeled 5′ × 5′ grid cell for each month during the 31-years of simulation (i.e. 372 concentrations per cell).

The value of *k* chosen for nano ZnO was 1.257∙10^−5^ s^−1^ and was obtained by fitting to data on nano ZnO sedimentation in river water measured by Keller et al. (2010) at 6-min intervals ranging between 0 and 400 min. This value corresponds to a half-life time of 15.3 h. Nano ZnO dissolution was assumed to have negligible impact on nano ZnO loss because data on nano ZnO dissolution in nanopore water measured over 800 h by Reed et al. (2012) indicates a dissolution rate of 3.21∙10^−8^ s^−1^ which is 391 times less than the chosen *k* value. Of course, the actual dissolution and sedimentation rates will vary depending on a large number or factors such as ENP coating, ENP size, ENP material and water pH, but this variability could not be reliably quantified due to lack of suitable data. The chosen nano ZnO *k* value is in the middle of a range of loss rates found for other ENPs in surface water under realistic conditions according to a review by Quik et al. (2011). We accounted for our uncertainty in the actual *k* value for nano ZnO by doing a worst-case simulation with a *k* of zero (Table 2).

The *k* value for nano Ag was based on the work of Kägi et al. (2011). They found that the dynamics of nano Ag concentrations in sludge and effluent of a pilot STP could be best explained by a mass balance model assuming that all nano Ag is sorbed to suspended and settled biosolids. They corroborated this finding with transmission electron microscopy images of sewage effluent showing that practically all nano Ag is sorbed to suspended biosolids. Therefore it was assumed that the nano Ag loss rate in surface waters, after being discharged by STPs, equals the settling loss rate of biosolids. This is about 1.26∙10^−4^ s^−1^ for the biosolids in the STP studied by Kägi et al. (2011), and this value was used here as the *k* for nano Ag. This corresponds to a modeled half-life time for nano Ag of 1.5 h. The resulting modeled loss of nano Ag is faster than loss rates measured for freely distributed nano Ag in freshwater samples (Chinnapongse et al., 2011, Quik et al., 2014). A worst-case simulation with a *k* of zero was also done to account for sewage discharges that had very limited or no treatment with biosolids (Table 2). In addition, this worst-case *k* assumes that the ENP dispersion in surface water is practically stable which could occur for example due the type of ENP surface coating or the surface water chemistry (Hammes et al., 2013). We have not treated sorption to suspended biosolids in effluent as ENP loss for two reasons: (1) This sorbed ENP may still be bio-available; (2) The nano Ag sorbed to biosolids is in equilibrium with the free nano Ag (Blaser et al., 2008) and thus remains a source of free nano Ag after emission from STPs. Dissolution losses of nano Ag were not modeled because results from Quik et al. (2014) show these are negligible compared to the modeled sedimentation losses.

### 2.3 Representation of model outputs

The median, average, and 90th percentile concentration were calculated for each cell, using all of the 372 monthly concentrations in the simulation period. These 372 concentrations vary as a result of temporal variability in hydrology (not emissions) which is a major driver because different hydrological variables (water discharge, abstractions, water levels) can vary with many orders of magnitude between different months. Temporal variability in other factors is accounted for by the modeled scenarios explained earlier. The 90th percentile concentration indicates the highest 10% of the concentrations in the water column that an aquatic organism in a particular grid cell will encounter during its life time. The median and average concentrations could be seen to represent long-term chronic exposure and accumulation of ENPs in tissues which may over time affect organism health.

These statistics were represented using maps and cumulative frequency curves. Maps indicated the spatial distribution of water concentrations, which allows an assessment of where critical levels might be exceeded. Cumulative frequency curves showed the percentage of surface waters where concentrations might exceed specific levels.

## 3 Results and discussion

The results presented in this section refer to the expected scenario, unless otherwise mentioned.

**Fig. 2:**
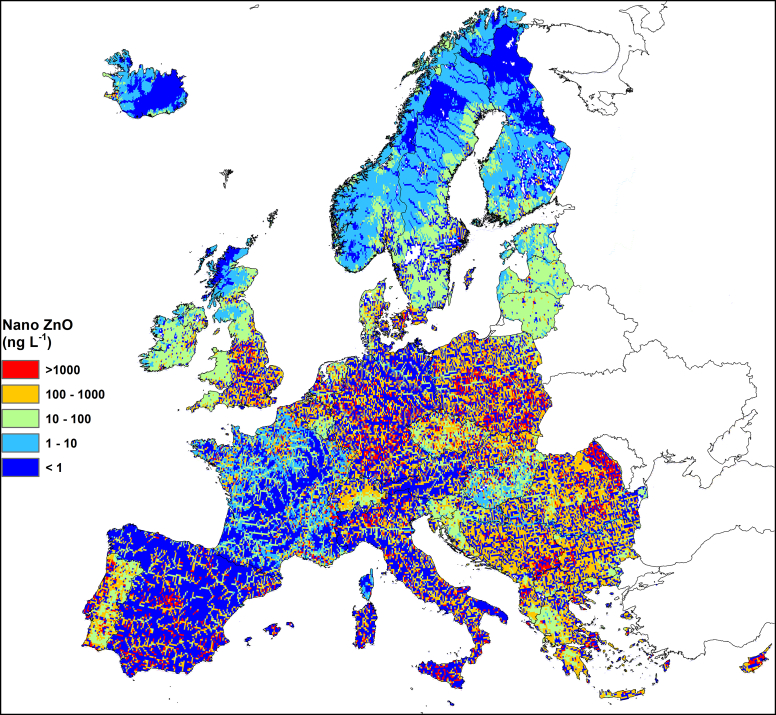
Map of 90th percentile expected nano ZnO concentrations.

**Fig. 3:**
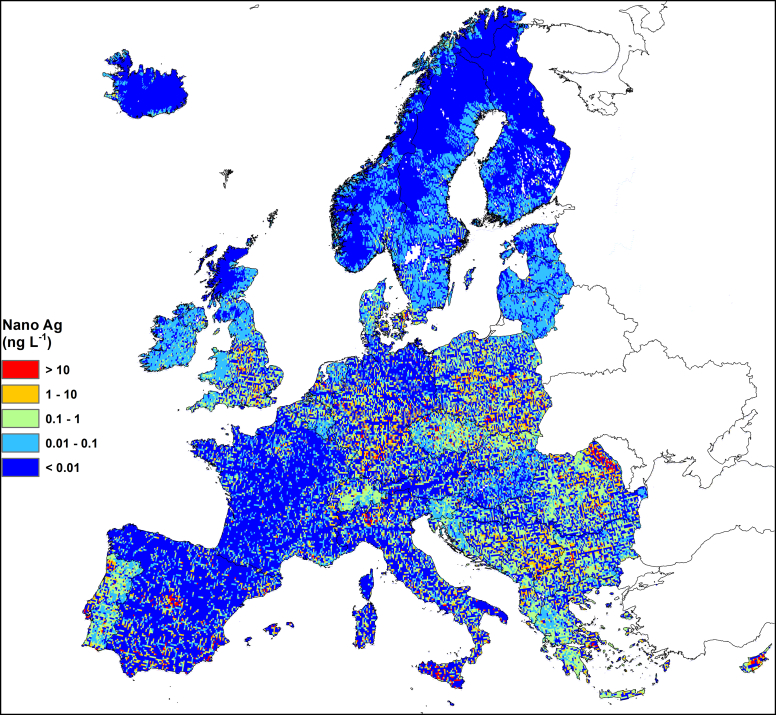
Map of 90th percentile expected nano Ag concentrations.

Looking across Europe, sparsely populated regions such as Iceland, Scandinavia, and Scotland show the lowest 90th percentile concentrations for both ENPs (Fig. 2, Fig. 3). The opposite can be seen in more densely populated regions. This pattern is slightly confounded by the tendency for 90th percentile concentrations in Eastern and Southern Europe to be increased by its relatively low water discharge during the summer months. The spatial pattern of ENP concentrations sometimes changes at country borders for two reasons: Differing completeness of effluent point data, and a differing spatial resolution of the census data underlying the population density map that was used to model *P*_cell_ where effluent point data was absent. Locally high concentrations are often found near large cities. Local modeled concentrations often do not change much in downstream direction if there is no confluence with a larger river reach. As a result, concentrations often are relatively constant in main stems of large rivers. This is especially clear for nano ZnO (Fig. 2), due to its smaller loss coefficient (*k*), making many main river stems look light blue to red on an otherwise dark blue background. Exceptions to this pattern are often caused by the presence of reservoirs causing concentrations in some large rivers to drop suddenly to relatively low levels. This is especially visible for nano ZnO (Fig. 2) in a number of large Spanish rivers, which sometimes have 15–40 km long stretches with very low concentrations just downstream of large reservoirs. Concentrations also tend to be higher further downstream in river systems because this is where STP discharges are more common.

**Fig. 4:**
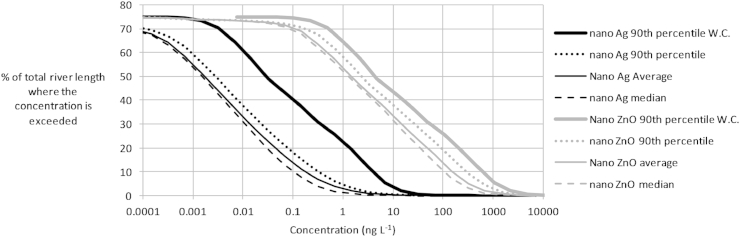
Cumulative-frequency curves of nano-particle concentrations in European rivers. The curves show the probability of encountering a river reach where a specific median, average, or 90th percentile concentration is exceeded. Worst-case curves are indicated with 'W.C.'.

It will be recalled that GWAVA predicts 372 concentrations in each cell in Europe using 31 years of monthly weather data. The temporal variability in each cell is summarized using the median, average, and 90th percentile concentrations. Half the river stretches have predicted long-term average concentrations exceeding 0.002 ng L^−1^ Nano Ag and 1.5 ng L^−1^ nano ZnO. In the 10% most exposed river reaches, concentrations in the 10% most exposed months exceed 0.3 ng L^−1^ for nano Ag and 300 ng L^−1^ for nano ZnO (90th percentile concentrations in Fig. 4). The median concentrations in the 10% most exposed river reaches were predicted to exceed 0.17 ng L^−1^ and 160 ng L^−1^ for nano Ag and nano ZnO, respectively. The average concentrations generally exceed the median concentrations, which is not surprising because the probability distribution of concentrations usually is skewed to the right. The 90th percentile concentrations of nano Ag in the worst-case scenario (lowest STP removal fraction and no loss in water column) were almost 13 times higher than in the expected scenario. For nano ZnO, they were about two times higher. This indicates that, while ENP concentrations are usually expected to be below the dotted lines in Fig. 4, they may be about a factor 2 to 13 higher locally (where STP removal fraction and water column loss have unlikely, but possible, values).

The 90th percentile concentration in individual grid cells is exceeded most often in July. The three-month period in which generally the highest concentrations are reached is June, July, and August (data not shown). In the rest of the year, the concentrations were generally lower and showed less temporal variation. Temporal variability in ENP concentrations can be mainly explained by temporal variability in modeled river water discharge (in the model, higher river water discharge usually causes higher dilution for ENPs from sewage effluent). The relatively high ENP concentrations from June to August are mostly due to the often low river discharge in these months. It is important to note that these three months often correspond with relatively high temperatures when feeding and reproduction of many aquatic organisms are likely to be higher. Also growth rates of pelagic communities are then likely to be higher due to higher solar radiation and longer water residence times (Hutchins et al., 2013). These factors could increase the ecological impact of the higher ENP concentrations during this period.

The modeled concentrations presented in this paper could be used as a basis for ecological risk assessment: the concentration maps could, for example, be used to indicate where a predicted no-effect concentration (PNEC) is exceeded, and the cumulative frequency curves could be used to indicate how frequently this occurs. PNECs of 2.19 μg L^−1^ for nano ZnO and 0.168 μg L^−1^ for nano Ag were, for example, estimated by Loureiro and van Gestel (2013). If these PNEC values were used then the PNEC would be exceeded in about 0.015% and 0.55% of European river reaches for nano Ag and nano ZnO respectively (Fig. 4). Obviously, if one would use lower PNECs then that would lead to higher modeled risk, and vice versa. A comprehensive assessment of risk is however beyond the scope of this paper.

### 3.1 Comparison with measurements and other models

ENP concentrations modeled using other methods and input data than those presented here vary widely. Most of these studies treat large areas and time periods as homogeneous (nano Ag: Boxall et al., 2007, Johnson et al., 2011, Gottschalk et al., 2009, Gottschalk et al., 2010, Mueller and Nowack, 2008) (nano ZnO: Boxall et al., 2007, Gottschalk et al., 2009), and therefore their 'lumped' concentration estimates can only be compared with this study after the modeled concentrations from this study are aggregated across space and time. Table 3 gives the comparison of these aggregated concentrations. The estimated concentrations in this study are rather low compared to most other estimates found in the literature, especially for nano Ag. The enormous variations in concentrations presented in these other estimates are due to differences between the modeled regions, assumed production volumes and market penetration factors (the assumed proportion of ENP-containing products in a modeled product category), as well as specific assumptions on elimination processes (especially removal rates in STPs and loss of ENPs from surface waters). The estimates in this study match reasonably well with the spatially and temporally explicit estimate made by Gottschalk et al. (2011), although in this study there was a larger difference between the two modeled scenarios. The latter difference is probably largely due to differences in scenario definition. The large range of predicted concentrations by other models and the absence of measured environmental concentrations make corroboration of results difficult. However there is no alternative at present.

**Table 3:** Comparison of modeled nano-particle concentrations with literature values. The concentrations from Gottschalk et al. (2011) are the 95th percentile across time and 85th percentile across space. The other literature concentrations are averages or medians across the model domain. Each concentration from this study was based on a scenario and aggregation method that matches as close as possible to the literature value to which it is compared.

ENP	Scenario	This study	Literature		
		Conc. (ng L^−1^)	Conc. (ng L^−1^)	Modeled area	Source
Ag	highest	0.016	100	UK	Boxall et al. 2007
Ag	expected	0.002	0.66	EU	Sun et al. 2014
Ag	highest	2.3	10	Switzerland	Gottschalk et al. 2011
Ag	lowest	0.16	8	Switzerland	Gottschalk et al. 2011
Ag	highest	0.024	0.55^a^	England & Wales	Johnson et al., 2014
ZnO	highest	2.6	760,000	UK	Boxall et al. 2007
ZnO	expected	1.5	90	EU	Sun et al. 2014
ZnO	highest	360	168	Switzerland	Gottschalk et al. 2011
ZnO	lowest	170	136	Switzerland	Gottschalk et al. 2011

a Concentration of colloidal Ag (which includes nano Ag).

It is however possible to make a comparison between measured and modeled sewage effluent concentrations (a state variable in GWAVA that had a country resolution in this study). Nano Ag concentrations of 0.3–14 ng L^−1^ have recently been measured in sewage effluents of nine STPs in Germany (Li et al., 2013). The expected nano Ag effluent concentration modeled for Germany (25 ng L^−1^) is above this measured range. This might mean that our modeled value for Germany is too high. However, it should be kept in mind that measured ENP concentrations are uncertain. Also, the limited number of measurements may have caused that their value range does not contain the expected effluent concentration for Germany, which is what GWAVA is trying to model. These considerations underline the uncertainty in the expected country-level sewage effluent concentrations, and their unmodeled (and largely unknown) variability within countries.

### 3.2 Remaining uncertainties

It is important to note that predictive models for chemicals can only be as good as their emission data, and currently information on ENP release to STPs from consumption and use is still limited. Official data for nano Ag and nano ZnO emissions is not available, and production volumes for nano Ag, on which ENP release to STPs in the EU can be based, vary by more than five orders of magnitude, thus indicating their uncertainty. Underestimated ENP production could have caused our method to underestimate aquatic exposure. Although we have used the most probable production volumes from Sun et al. (2014), their raw production data indicates that the production of nano ZnO and nano Ag could be about 1.6 and 4.3 times higher, respectively. Such higher production volumes would result in aquatic concentrations that are also approximately a factor 1.6–4.3 higher. Another source of possible underestimation of ENP input into our model could be ENP leaching from landfills which, especially over time, may become an important factor.

This study only considers the ENP exposure from losses during the use of household products. This means that there could be locally underestimated ENP concentrations, for example near the small minority of STPs serving ENP related industry.

This paper may underestimate the spatial variability in concentrations because the calculation of loading to surface waters assumes that the ENP emission per person to sewerage is constant across all Europe, whereas in reality it may vary with factors such as income and product preference. This assumption of spatially constant ENP emission per person was driven by lack of suitable data. Possible underestimation of spatial variability in our predicted concentrations is also driven by data limitations regarding two important fate parameters: Firstly, the surface water loss coefficient (*k*) had to be assumed constant. Secondly, removal in STPs (*STP*_rem_) had to be assumed constant because available data was insufficient for deriving a relation between STP treatment level (e.g. primary, secondary) and ENP removal. Similarly, lack of suitable spatial data was the reason why it was impossible to consider that storm water in certain residential areas does not undergo treatment.

Risk estimates based on ENP exposure estimates such as those in this study need to be done with caution. For example, underestimation of total ENP induced risk may occur if the conversion of nano Ag to dissolved silver ions is not considered: it is widely accepted that free silver ions are the most toxic form of silver (e.g. Bilberg et al., 2012). On the other hand, overestimation of risk may result if one does not consider that a proportion of the modeled ENPs have been transformed after release from households, especially by phosphatization and sulfidization (e.g. Kim et al., 2010, Kägi et al., 2011, Lombi et al., 2012). Not only are these transformation processes likely to reduce hazard in surface waters, they could also accelerate aggregation and then sedimentation (Dale et al., 2013, Ma et al., 2013a), and hence reduce risk in surface waters. Also, they could reduce nano-particle specific risks by changing a proportion of the original particles to non-nano sizes. There was not sufficient information in the current literature to reliably quantify the impact of these transformation processes on the scale of this study. This study may also have overestimated the exposure to ENPs where the ENPs are less bio-available due to (temporary) sorption to suspended matter. Finally, the mismatch between the temporal resolution of our predicted concentrations (monthly) and the duration of typical toxicity tests (days) may lead to underestimation of risk. In future work, we hope to resolve the latter issue by using a daily time step.

Data availability at the European scale was insufficient to reliably model ENP concentrations in the river bottom sediments. However, it would be important to attempt this in the future after more detailed data has become available because more localized modeling studies (e.g. Praetorius et al., 2012) indicate that ENP concentrations in sediments can be a million times higher than in the overlying water.

We have chosen for two deterministic model runs ('worst-case' and 'expected') using two different possible values for the two most sensitive model parameters. While this is useful for risk assessment (The worst-case run may help to indicate where all risk can be excluded, and the expected run may help to indicate where risk is unlikely), it does not give the probabilities of all possible environmental concentrations. We decided for those two deterministic model runs because our study aims at supporting risk assessment. Also the large number of runs involved in a stochastic simulation would require too much computer time and generate excessive amounts of output data. However, we hope to make a stochastic simulation possible in the future.

As mentioned previously, uncertainty in the model parameters may result in a range of possible concentrations. Indeed, uncertainty in the two most sensitive model parameters (*STP*_rem_ and *k*) may result in concentrations that are a factor 2 to 13 higher than the expected values. Generally, data available in the literature on loss rates of ENPs in surface waters is still very limited, which affects the uncertainty in the results presented here.

## 4 Conclusions

The GWAVA model was used to simulate the exposures resulting from current levels of production and use of nano Ag and nano ZnO in Europe. The simulation used a representative 31-year period of monthly weather data. In the 10% most exposed river reaches, concentrations in the 10% most exposed months exceed 0.3 ng L^−1^ for nano Ag and 300 ng L^−1^ for nano ZnO. These higher concentrations were typically found in Eastern and Southern Europe, near large cities, and further downstream in river systems.

The large range of predicted concentrations by other models and the absence of measurements made validation difficult. Nevertheless it is argued that the presented model is an improved method for ENP risk assessment as it represents the topography of the dominant real-world sources and sinks, and because it represents non-equilibrium concentrations in surface water, as opposed to current standard risk-assessment methods.

## Footnotes

1 Symbol ' indicates arc-minute; a unit of length used in mapping.
