# Supplementary material for: Nano silver and nano zinc-oxide in surface waters – Exposure estimation for Europe at high spatial and temporal resolution
Source: Environ Pollut. 2015 Jan;196:341–9. doi: 10.1016/j.envpol.2014.10.022 (PMC4270461; doi:10.1016/j.envpol.2014.10.022)
Supplement: Supplementary file 1 [file mmc1.docx]

Nano silver and nano zinc-oxide in surface waters - Exposure estimation for Europe at high spatial and temporal resolution

Egon Dumont^a,1^, Andrew C. Johnson^a^, Virginie D.J. Keller^a^, Richard J. Williams^a^

^a^: Centre for Ecology & Hydrology (CEH), Centre for Ecology & Hydrology, Maclean Building, Benson Lane, Wallingford, OX10 8BB, United Kingdom

^1^: Corresponding author.

E-mail address: egdu@ceh.ac.uk

Telephone number: +44 1491 692291

Fax number: +44 1491 692430**Table S1** Literature data used in modeling nano Ag and nano ZnO removal in sewage treatment works

|  | **Nano Ag** | **Nano ZnO** |
| --- | --- | --- |
| Li et al. (2013) | 95% (Field studies on 9 STPs) | NA |
| Studies run by Work Package 2 of EU project NanoFate | 99% (pilot STP study, 2 separate runs) | NA |
| Lombi et al. (2012) | NA | 82% (based on nano ZnO STP sediment K_d_ of 1280 from Lombi et al. (2012) and assumed 3.5 g/L sediment in STP) |
| Sun et al. (2014) | 96% (Calculation based on modeling/review) | 88% (Calculation based on modeling/review) |
| Environment Agency (2011) – general removal for Zn applied in environmental concentration estimations for discharges from STPs | NA | 81% |
| Kaegi et al. (2011) | 95% to sludge (1 pilot STP) | NA |
| Schlich et al. (2013) | (1 pilot STP) 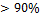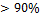 | NA |
| Lombi et al. (2013) | 85% (large batch microcosms) | NA |
| Tiede et al. (2010) | 93% (batch microcosms) | NA |
| Park et al. (2013) | >90% (lab pilot STP) | NA |
| Kiser et al. (2010) | 97% (batch microcosms) | NA |
| Wang et al. (2012) | 88% (pilot SBR test in lab) | NA |

# References

Kägi, R.; Voegelin, A.; Sinnet, B.; Zuleeg, S.; Hagendorfer, H.; Burkhardt, M., et al., 2011. Behavior of Metallic Silver Nanoparticles in a Pilot Wastewater Treatment Plant. Environ. Sci. Technol. 45, 3902-3908.

Kiser, M.A., Westerhoff, P.K., Ryu, H., Benn, T., 2010. Occurrence and fate of engineered nanomaterials in wastewater treatment plants. Abstracts of Papers of the American Chemical Society 240.

Li, L., Hartmann, G., Doblinger, M., Schuster, M., 2013. Quantification of nanoscale silver particles removal and release from municipal wastewater treatment plants in Germany. Environ. Sci. Technol. 47, 7317-7323.

Lombi, E., Donner, E., Tavakkoli, E., Turney, T.W., Naidu, R., Miller, B.W., Scheckel, K.G., 2012. Fate of Zinc Oxide Nanoparticles during Anaerobic Digestion of Wastewater and Post-Treatment Processing of Sewage Sludge. Environ. Sci. Technol. 46, 9089-9096.

Lombi, E., Donner, E., Taheri, S., Tavakkoli, E., Jamting, A.K., McClure, S., Naidu, R., Miller, B.W., Scheckel, K.G., Vasilev, K., 2013. Transformation of four silver/silver chloride nanoparticles during anaerobic treatment of wastewater and post-processing of sewage sludge. Environ. Pollut. 176, 193-197.

Park, H.J., Kim, H.Y., Cha, S., Ahn, C.H., Roh, J., Park, S., Kim, S., Choi, K., Yi, J., Kim, Y., Yoon, J., 2013. Removal characteristics of engineered nanoparticles by activated sludge. Chemosphere 92, 524-528.

Schlich, K., Klawonn, T., Terytze, K., Hund-Rinke, K., 2013. Hazard assessment of a silver nanoparticle in soil applied via sewage sludge. Environ. Sci. Eur. 25, 17.

Sun, T.Y., Gottschalk, F., Hungerbuhler, K., Nowack, B., 2014. Comprehensive probabilistic modelling of environmental emissions of engineered nanomaterials. Environ. Pollut. 185, 69-76.

Tiede, K., Boxall, A.B.A., Wang, X.M., Gore, D., Tiede, D., Baxter, M., David, H., Tear, S.P., Lewis, J., 2010. Application of hydrodynamic chromatography-ICP-MS to investigate the fate of silver nanoparticles in activated sludge. J. Anal. Atom. Spectrom. 25, 1149-1154.

Wang, Y., Westerhoff, P., Hristovski, K.D., 2012. Fate and biological effects of silver, titanium dioxide, and C-60 (fullerene) nanomaterials during simulated wastewater treatment processes. J. Hazard. Mater. 201, 16-22.
